# Group-wise sparse coding for the discovery of functional connectivity-based biomarkers in children and adolescents with autism spectrum disorder

**DOI:** 10.3389/fpsyt.2026.1743755

**Published:** 2026-07-23

**Authors:** Yonglu Wang, Zhangliang Ma, Zhiyi Wang, Xinyue Xu, Zhengwang Xia, Huihui Zhang, Jiuping Zhang, Yang Zong, Xiaoyan Ke, Yun Li

**Affiliations:** 1 Child Mental Health Research Center, The Affiliated Brain Hospital of Nanjing Medical University, Nanjing, China; 2 School of Computer Information and Engineering, Changzhou Institute of Technology, Changzhou, China; 3 Faculty of Sports and Exercise Science, University of Malaya, Kuala Lumpur, Malaysia

**Keywords:** autism spectrum disorder, children and adolescent, dictionary learning and sparse coding, empathy, functional brain connectivity

## Abstract

**Background:**

Autism spectrum disorder (ASD) is currently diagnosed through behavioral observations and evaluations, but there is still a lack of objective and consistent biomarkers. Children and adolescents with ASD exhibit impairments in advanced social, emotional, and cognitive functions, such as a lack of empathy. In general, the concept of empathy encompasses several socio-emotional and cognitive components based on interacting brain circuits. Identification of disease-related biomarkers at the brain network level could provide a crucial avenue for advancing ASD imaging research and improving diagnostic accuracy.

**Methods:**

This study examined 80 individuals with ASD aged 6–16 years old and 50 matched control subjects, using resting-state functional magnetic resonance imaging and clinical psychological assessment datasets. Specifically, a set of functional brain networks was constructed using dictionary learning and sparse coding (DLSC) in a group-wise manner. Then, the localized common functional brain networks from both the ASD and matched control groups were automatically decomposed into a set of regions of interest (ROIs) for further functional connectivity analyses.

**Results:**

Using the derived functional connectivity matrix, we investigated three parameters, namely, correlation, partial correlation, and tangent embedding, to differentiate participants with ASD from control subjects. We achieved classification accuracies of 95%, 100%, and 100%, respectively, indicating that the proposed DLSC method could extract representative and characteristic brain ROI atlases for both ASD and control participants. Further analysis of functional connectivity results showed that ASD participants had multiple atypical connections, especially those connecting the left inferior temporal and left inferior parietal regions, which belonged to the temporoparietal junction (TPJ), and connections related to the right insula and anterior cingulum, which belonged to the salience network (SN). Together, our results suggest that individuals with ASD exhibited a lower empathy capability than control subjects.

**Conclusion:**

Our results suggest that DLSC can effectively extract robust brain ROI atlases. Functional connectomes with high differentiation powers were mainly distributed within the brain networks of SN, social brain networks (SBNs), and the theory of mind (ToM) network (including the TPJ hub). Children and adolescents with ASD exhibited lower empathy capabilities than control subjects, which may be attributed to dysfunctions in the salience and social brain networks.

**Clinical Trial Registration:**

https://www.chictr.org.cn, identifier ChiCTR-ROC-17012877.

## Introduction

Autism spectrum disorder (ASD) is a neurodevelopmental condition characterized by persistent deficits in social communication and interaction across multiple contexts, along with restricted, repetitive patterns of behavior, interests, or activities (1). A lack of empathy is one of the core social communication impairments observed in individuals with ASD (2, 3). Empathy is defined as the ability to recognize, understand, and share the emotional states of others, serving as the foundation for sincere and mutually beneficial relationships (4). Numerous definitions of empathy encompass a broad range of emotional states. The main types of empathy include cognitive empathy, affective empathy, and somatic empathy (5). In this study, we examined both affective and cognitive aspects of empathy.

Because ASD is a neurodevelopmental condition associated with advanced cognitive impairments, characterizing functional differences between participants with ASD and control subjects can provide a direct and efficient way to identify the underlying mechanisms of ASD. Functional activity across brain regions that operate simultaneously is considered to constitute the brain network, which can be measured using intrinsic functional connectivity (FC) (6). FC analysis involves identifying brain regions whose blood oxygenation level-dependent (BOLD) signals obtained from resting-state functional magnetic resonance imaging (rs-fMRI) co-fluctuate (7). These co-fluctuating regions are considered to form a brain network supporting a specific cognitive or mental processing task. Functional MRI (fMRI) studies have reported that the theory of mind (ToM) involves the medial prefrontal cortex (mPFC), temporoparietal junction (TPJ), middle temporal gyrus, temporal pole, and posterior cingulate cortex/precuneus (8–10). Other studies using rs-fMRI have reported that brain regions within the default mode network (DMN) overlap with the brain regions associated with ToM. Thus, using rs-fMRI-derived FC may provide quantitative insights into the social-cognitive capabilities of individuals with ASD (11–14). For example, Assaf et al. (11) reported that FC values in specific brain regions were highly correlated with Social Responsiveness Scale scores in a group of individuals with ASD. Weng et al. (13) reported that FC strength between the posterior cingulate cortex and the temporal lobe was negatively correlated with social impairment based on the Autism Diagnostic Interview–Revised (ADI-R) system. These studies have suggested the potential value of using rs-fMRI to investigate complex cognitive functions in individuals with ASD.

Prior resting-state fMRI studies of ASD have characterized functional connectivity across whole-brain scales (15–17), including distributed correlational networks (18, 19) and graph-theoretical measures (20, 21). However, the majority of studies have focused on individual brain regions or a limited number of preselected brain regions or networks. Increasing evidence indicates that human brain function arises from the concurrent interaction of multiple distributed, connectome-scale neural networks (22–24). Identifying such networks in ASD necessitates defining a set of functionally meaningful, group-consistent regions of interest (ROIs) that serve as a common reference framework for cross-subject comparisons. This challenge extends beyond spatial normalization to a standard template space, which is routinely addressed in rs-fMRI studies, and instead concerns the functional validity of the chosen parcellation scheme (25). Widely used atlas-based approaches, including anatomical templates such as the Anatomical Automatic Labeling (AAL) atlas, which disregards functional boundaries, and functional atlases such as the seven-network parcellation (26), which is derived from large cohorts of neurotypical adults, impose a fixed, *a priori*, parcellation that may not fully capture the functional network topology specific to atypical developmental populations, such as children and adolescents with ASD. Consequently, standard atlases may lack sensitivity to subtle, cohort-specific variations in functional architecture.

**Figure 1:**
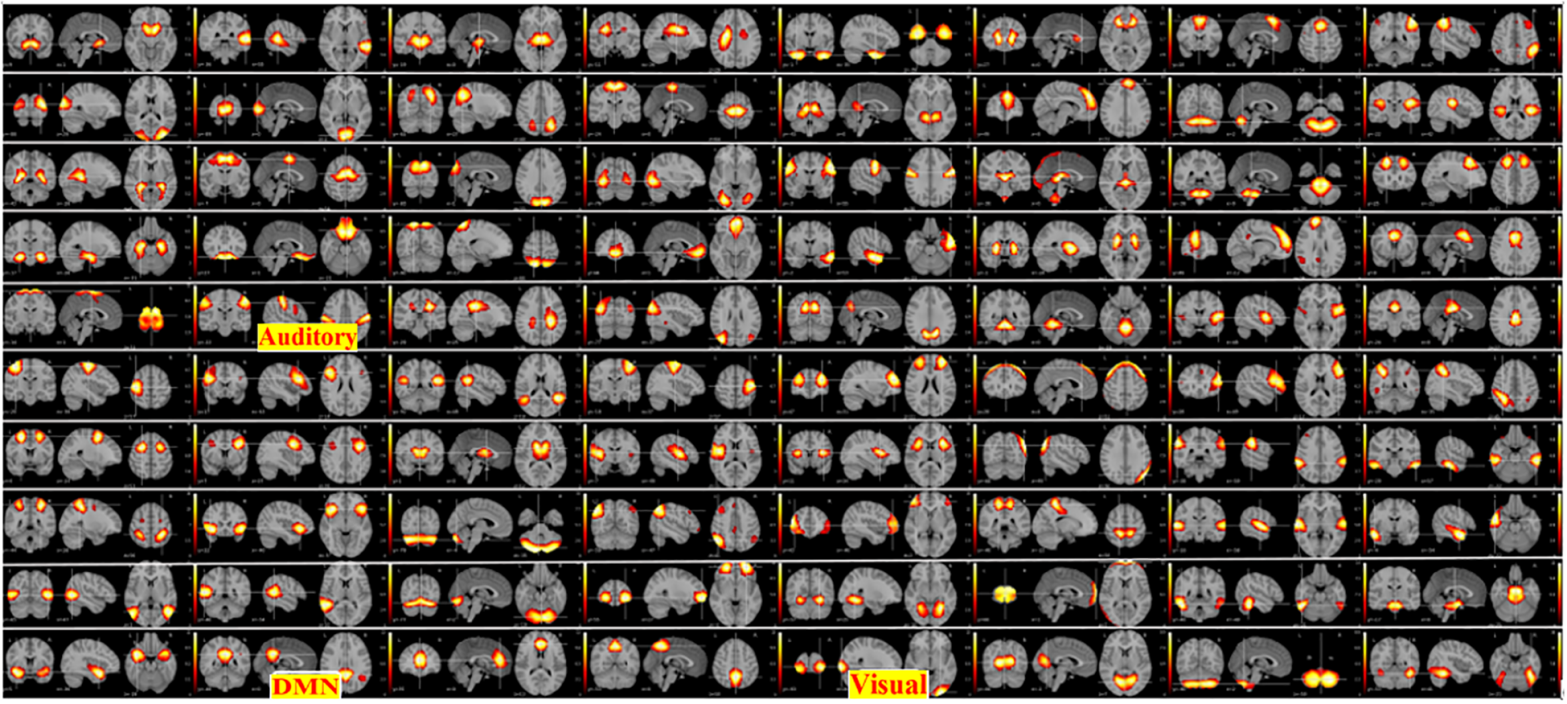
Visualization of the 80 common functional brain networks learned using dictionary learning and sparse coding (DLSC). Each subpanel represents one dictionary atom mapped onto the brain volume, showing the spatial pattern of a distinct functional network. Representative canonical networks are highlighted with white color borders and labels: the DMN encompassing the medial prefrontal cortex, posterior cingulate cortex/precuneus, and bilateral angular gyri; the visual network comprising the primary and associative visual cortices along the calcarine sulcus and occipital pole; and the auditory network localized to the bilateral superior temporal gyri. These networks are spatially consistent with established resting-state network templates and demonstrate the ability of DLSC to decompose whole-brain signals into functionally meaningful and neuroanatomically interpretable components. DMN, default mode network; L, left; R, right.

To address this gap, we employed a data-driven, group-wise dictionary learning and sparse coding (DLSC) approach that learns a common set of functional network components directly from the aggregated resting-state fMRI data of both ASD and typically developing control (TDC) participants. Unlike seed-based methods, which depend on subjective seed selection (18), graph-theoretical approaches, which are sensitive to the chosen parcellation scheme (20), and independent component analysis (ICA), which does not enforce sparsity (27–29), sparse coding imposes an ℓ_1_ regularization constraint that aligns with the intrinsically sparse nature of neural activity, yielding spatially focal and minimally overlapping functional networks (30–32). In the DLSC framework, group-wise, temporally concatenated, whole-brain BOLD signals are factorized into a shared dictionary and participant-specific sparse coefficient vectors. Because the dictionary is learned jointly from both groups, it establishes inherent inter-subject correspondence and a common reference space, enabling direct, unbiased statistical comparisons of functional connectivity while adaptively capturing the intrinsic functional organization of the specific cohort under investigation. We note that DLSC-derived networks are not proposed as a replacement for well-validated atlases (26). As shown in **Figure 1**, the learned components exhibit strong spatial convergence with canonical networks, including the default mode, visual, and auditory systems, while additionally revealing finer-grained parcellations (e.g., anterior and posterior DMN subsystems and dorsal and ventral insular divisions) that may reflect cohort-specific functional architecture and the enhanced spatial resolution conferred by the sparsity constraint.

We applied this framework to an rs-fMRI dataset comprising 50 Typically developing controls (TDCs) and 80 individuals with ASD aged 6−16 years. For each participant, three types of functional connectivity measures, namely, correlation, partial correlation, and tangent embedding (33), were computed between all pairs of DLSC-derived ROIs and served as input features to ℓ_2_-penalized linear support vector machine (SVM) classifiers (34) for distinguishing ASD from TDC participants. Classification performance was evaluated using leave-one-out cross-validation (LOOCV) because of the limited sample size. The most discriminative functional connections were identified via two-sample t-tests as candidate connectivity-based biomarkers for ASD, and their associations with empathic ability were subsequently assessed using the Griffith Empathy Measure parent ratings (GEM-PR) (35).

## Materials and methods

### Subject data acquisition

#### Ethics statement

This study was approved by the Medical Ethics Committee of the Brain Hospital Affiliated to Nanjing Medical University (KY043). The parents or legal guardians of the participants were informed of the study objectives and detailed procedures of the investigation and signed written informed consent before the study was initiated. The study was performed in accordance with the Declaration of Helsinki. Children with ASD were selected from the ASD-specific disease cohort at the Child Mental Health Research Center, Affiliated Brain Hospital of Nanjing Medical University. Recruitment was conducted from December 2020 to June 2024, and all methods were performed in accordance with relevant guidelines and regulations.

### Phenotypes of participants

Typically, children with ASD exhibit specific profiles when assessed using the Wechsler Intelligence Scale for Children (WISC), the most commonly used test for assessing intelligence and intelligence quotient (IQ). All participants had IQ scores above 70 points (36, 37). In this study, a total of 130 participants were enrolled, including 80 with ASD and 50 TDC subjects. The participants were matched for sex, age, and IQ. Diagnosis for ASD was based on the DSM-5. Participants with systemic disease, a family history of head injury, genetic syndromes, neurological disorders, or psychiatric illness were excluded from the study. The detailed clinical and demographic data for all individuals are presented in **Table 1**.

**Table 1:** Demographic and clinical characteristics of participants with ASD and control participants.

Variables	ASD group	TDC group	*t/χ^2^*	*p*
Sex				
Male	69	37		
Female	11	13		
Age (years)	9.503 ± 2.512	10.022 ± 3.124	-1.041	0.300
IQ	103.325 ± 20.968	108.860 ± 17.660	-1.553	0.123
ADI-R				
Social interaction	14.750 ± 6.778	–	–	
Language	9.438 ± 4.847	–	–	
Stereotyped behavior	4.500 ± 2.755	–	–	
ADOS				
Communication	4.388 ± 2.242	–	–	
Social interaction	7.538 ± 2.908	–	–	
Imaging/creativity	1.125 ± 0.986	–	–	
Stereotyped behavior and imitation	1.425 ± 1.281	–	–	
CARS	30.338 ± 3.094			
GEM-PR total	10.963 ± 18.025	30.020 ± 19.719	-5.655**	**0.000**
GEM-PR cognitive	3.050 ± 10.771	24.540 ± 17.655	-8.627**	**0.000**
GEM-PR affective	12.038 ± 16.282	17.840 ± 13.196	-2.121*	**0.036**
Head motion parameters				
Mean FD (mm)	0.18 ± 0.07	0.16 ± 0.06	1.672	0.097
Max FD (mm)	0.72 ± 0.31	0.68 ± 0.28	0.742	0.459
Volumes censored (%)	8.45 ± 5.62	7.38 ± 4.91	1.103	0.272
Retained volumes	129.96 ± 8.12	131.51 ± 7.45	-1.152	0.252
Total volumes acquired	142	142	—	—

IQ, intelligence quotient; ADI-R, Autism Diagnostic Interview–Revised; ADOS, Autism Diagnostic Observation Schedule; ASD, autism spectrum disorder; TDC, typically developing control; CARS, Childhood Autism Rating Scale; GEM-PR, Griffith Empathy Measure-Parent Ratings; FD, framewise displacement. ^*^p < 0.05, ^**^p < 0.01, ^***^p < 0.001. Bold values indicate statistical significance.

### Clinical psychological assessments

General condition survey: A self-compiled questionnaire was used to collect general demographic data, birth history, disease history, and family history from all participants.

Clinical assessment: (1) The Autism Diagnostic Interview-Revised (ADI-R) (38) and the Autism Diagnostic Observation Schedule, Second Edition (ADOS-2) (39) were administered as standardized, semi-structured instruments to characterize the autistic phenotype of the individual and to support the clinical diagnostic process. The clinical diagnosis of ASD was established by licensed child and adolescent psychiatrists based on a comprehensive evaluation integrating developmental history, direct behavioral observation, cognitive and language assessments, and DSM-5 diagnostic criteria. The ADI-R and ADOS-2 served as standardized supporting measures rather than standalone diagnostic instruments. The ADI-R is a widely used semi-structured diagnostic interview administered to caregivers that focuses on three domains: communication (verbal communication [VC] and non-verbal communication [NVC]), reciprocal social interaction (RSI), and repetitive behavior and stereotyped patterns (RBSP). Each item is scored from 0 (no impairment) to 2–3 (very severe delay/deviance). To satisfy the ADI-R criteria for autism, a child must score above the cutoff in all three domains and have exhibited developmental concerns before 3 years of age. The ADI-R cutoff scores were 10 for RSI, 7 for NVC, 8 for VC, and 3 for RBSP. The ADOS-2 is a semi-structured, standardized assessment of communication, social interaction, and play (or imaginative use of materials) for individuals suspected of having autism or other pervasive developmental disorders. It consists of five modules, each tailored to individuals with different developmental and language levels: the Toddler Module (for children 12−30 months of age with limited expressive language), and Modules 1–4 (for individuals ranging from non-verbal children to verbally fluent adolescents and adults). In the present study, Modules 1–4 were administered according to each participant’s chronological age and expressive language level. Modules 1–3 additionally provide a calibrated severity score (comparison score), which indexes the level of autism spectrum-related symptoms relative individuals with ASD of the same age and comparable language skills. All ADOS-2 administrations were conducted by licensed child and adolescent psychiatrists who had completed standardized research reliability training. (2) The Childhood Autism Rating Scale (CARS) was used as part of the clinical evaluation of the child. The CARS is a diagnostic assessment used to observe children together with parental observations. A total score > 30 is indicative of autism. (3) The Wechsler Intelligence Scale for Children–China Revised (WISC-CR) is an IQ determination scale that includes a total of 12 subtests. (4) The Griffith Empathy Measure Parent Ratings (GEM-PR) (35) was used to assess empathy in both groups of children. The Griffith empathy test is a questionnaire with 23 items for children and adolescents of comparable mental ability. It is scored from −4 to 4, and each item is easy to understand and has demonstrated high reliability in the Chinese population. For clinical and healthy populations, the Griffith empathy test has been widely used to measure empathy because of its good reliability and validity across individuals of different ages and sexes. The test is divided into two dimensions: cognition and affective empathy. Cognitive empathy refers to the ability to look at and understand emotions from the perspective of others. The definition of affective empathy is the experience of another person’s emotional state. This questionnaire was completed by the parents.

In this study, standardized clinical assessments, including the CARS, ADI-R, ADOS, and WISC-CR, were conducted by licensed child and adolescent psychiatrists for both groups.

### Magnetic resonance imaging data acquisition and parameters

MRI data were acquired using a 3.0T clinical scanner (Siemens Medical Systems; Munich, Germany) equipped with a birdcage gradient head coil. Cross-sectional T2-weighted imaging (T2WI) was first performed for each case to ensure anatomical positioning and to exclude individuals with the visible organic nervous system diseases. High-resolution three-dimensional T1-weighted structural images were acquired using a magnetization-prepared rapid gradient echo (MPRAGE) sequence with the following parameters: repetition time (TR) = 2530 ms, echo time (TE) = 3.34 ms, slice thickness = 1.33 mm, flip angle = 7°, inversion time (TI) = 1100 ms, field of view (FOV) = 256 × 256 mm², acquisition matrix = 256 × 192, and total acquisition time = 8 min 7 s. The scanning plane was oriented parallel to the line connecting the genu and splenium of the corpus callosum, covering the brain from the vertex to the inferior cerebellum, with a total of 128 slices. The acquisition parameters for the rs-fMRI scans were as follows: gradient echo pulse sequence, echo planner imaging sequence, TR = 2,000 ms, TE = 30 ms, matrix = 64 × 64, flip angle = 90°, field of view = 240 × 240 mm^2^, slice thickness = 4 mm, slices = 30, slice gap = 0 mm, scanning time = 284 s, and voxel size = 3.75 × 3.75 × 4.0 mm.

### The rs-fMRI data processing procedures

#### Overview

**Figure 2:**
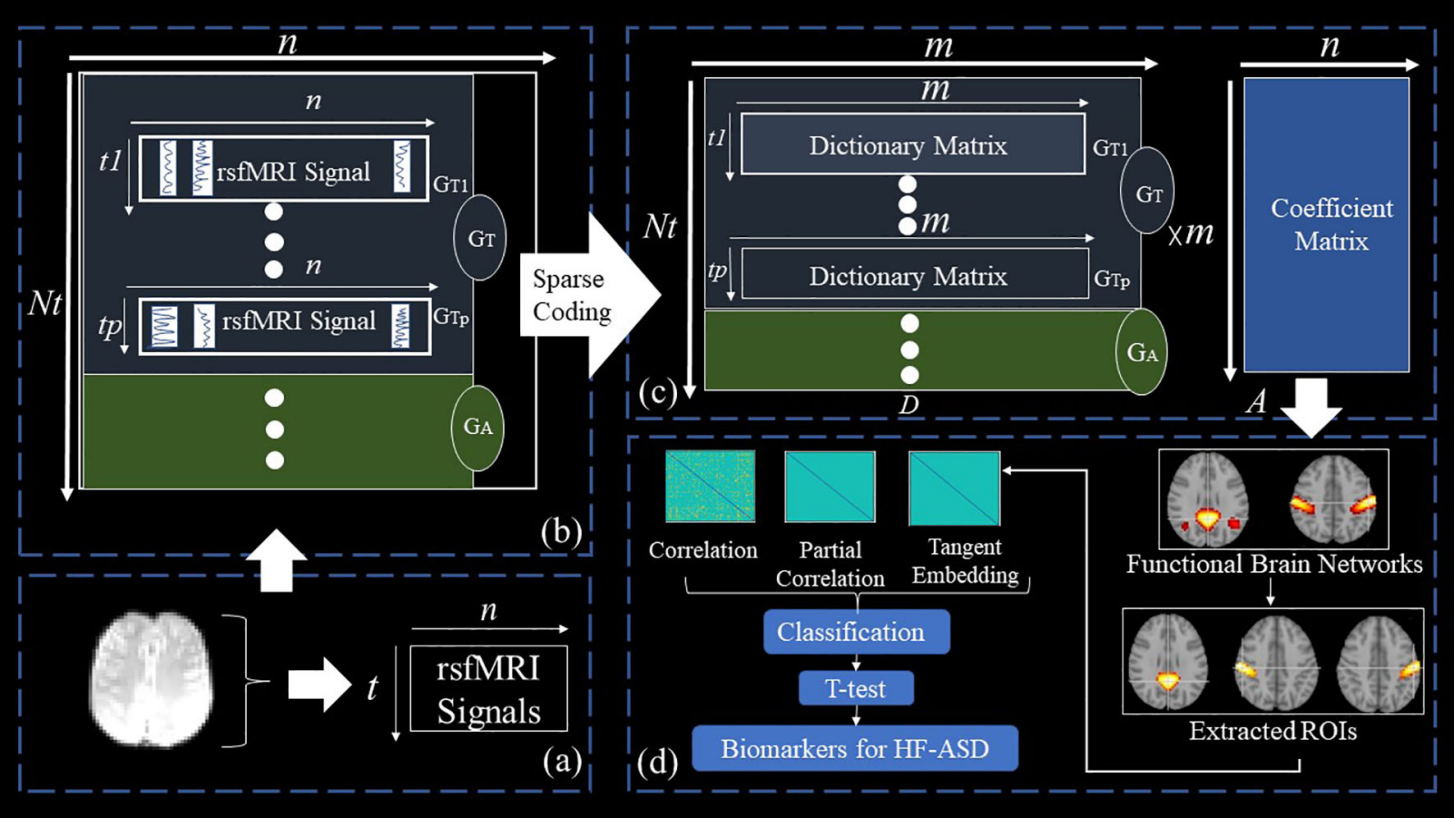
Computational pipeline for deriving functional connectivity-based biomarkers using group-wise dictionary learning and sparse coding (DLSC). G_T_ represents the development control group, and G_A_ represents the autism spectrum disorder group. **(a)** Whole-brain resting-state functional magnetic resonance imaging (rs-fMRI) signals of each individual were aggregated into a single signal matrix, in which each row represents the whole-brain rs-fMRI data at a single time point, and each column represents the time series of a single voxel. A unified mask in the Montreal Neurological Institute space guided the signal extraction. **(b)** The rs-fMRI signals from all individuals in both groups were pooled and arranged into a large matrix along the time dimension. **(c)** The group-wise DLSC framework. **(d)** The process of regions of interest extraction, functional connectivity analysis, classification, and the two-sample *t*-test. ROI, region of interest; ASD, autism spectrum disorder.

The overall computational pipeline is illustrated in **Figure 2**. After preprocessing (described in the next section), the rs-fMRI signals of all voxels within the whole brain were extracted and arranged in a single signal matrix for each individual (**Figure 2a**). Then, the signal matrices from all individuals in both groups (TDC and ASD) were aggregated along the time dimension to generate a large data matrix, which served as the input to the group-wise DLSC framework (**Figure 2b**). **Figure 2c** illustrates the framework of the group-wise DLSC approach, where common functional brain networks of the two groups were sparsely learned and mapped from rows of the coefficient matrix *A*. Next, the common brain networks were divided into a set of ROIs by labeling each region based on the presence of unique features (33). Using the extracted ROIs as nodes, three different connectivity measures were calculated to identify functional relationships between brain regions (**Figure 2d**). Finally, $l_{2}$-penalized linear SVM classifiers were used to evaluate the effectiveness of the group-wise DLSC framework for extracting brain ROIs. Then, functional connectivity-based biomarkers for ASD were further derived by performing a two-sample *t*-test (**Figure 2d**).

#### Preprocessing

Preprocessing of the rs-fMRI data was performed using the FSL toolbox (40). The preprocessing pipeline consisted of the following steps: (1) skull removal using the Brain Extraction Tool (BET); (2) rigid-body motion correction using the MCFLIRT command, with a 4-mm (voxel-level) motion parameter search to identify and remove micro head motions (41); (3) slice timing correction; (4) spatial smoothing with a 6-mm full width at half maximum (FWHM) Gaussian kernel; (5) temporal pre-whitening to account for serial autocorrelations in the BOLD time series; (6) removal of linear and quadratic temporal drifts (global drift removal); (7) volume censoring (scrubbing) to further mitigate motion-related artifacts; and (8) linear registration to the Montreal Neurological Institute (MNI) 152 standard brain template space using FLIRT and FEAT.

Regarding motion correction, we implemented a rigorous multi-step procedure. Following initial rigid-body realignment with MCFLIRT, framewise displacement (FD) was calculated as the sum of the absolute values of the six realignment parameter derivatives (three translational and three rotational) at each time point (42, 43). Volume censoring (scrubbing) was performed by identifying volumes with FD values exceeding 0.5 mm; these flagged volumes, together with one preceding and two subsequent volumes, were excluded from subsequent analyses to minimize the residual influence of motion artifacts (44). Participants with more than 30% of total volumes censored were excluded from the study. Following manual visual inspection, all 80 participants with ASD and 50 TDC participants met this retention criterion. Motion metrics, including mean FD and the number of retained volumes after scrubbing, were computed for each participant and compared between groups. No significant between-group differences were observed in any motion metric (all *p* > 0.05; see **Table 1**), confirming that the two groups were adequately matched with respect to in-scanner head motion.

Following registration, nuisance signal regression was performed to reduce physiological noise. Mean BOLD time series were extracted from individually segmented white matter and ventricular cerebrospinal fluid (CSF) masks, which were generated from the T1-weighted structural images using the FSL FAST automated segmentation tool (40, 45). These white matter and CSF signals, along with the six rigid-body motion parameters (three translational and three rotational) and their temporal derivatives, were included as nuisance regressors in a general linear model and regressed out of the preprocessed rs-fMRI data. Notably, global signal regression (i.e., regression of the mean whole-brain or gray matter signal) was intentionally not performed. This decision was based on the following considerations: (1) global signal regression remains methodologically controversial, as it can artificially introduce negative correlations and distort interregional connectivity patterns (46–48); (2) the global signal may carry neurally meaningful information, particularly in clinical populations such as individuals with ASD, in whom global signal topography has been reported to differ from that of neurotypical controls (49, 50); and (3) the combination of rigorous volume censoring (scrubbing) with white matter and CSF nuisance regression provides substantial mitigation of motion-related and physiological artifacts while preserving potentially informative gray matter signal variance (44, 51). After preprocessing, binary masks indicating voxels with non-zero rs-fMRI signals were generated for all individuals.

### DLSC

The dictionary learning and sparse coding method has proven effective for modeling functional brain networks (32, 52–55). Therefore, we employed this method in our study to generate functionally consistent networks and their nodes for constructing connectomes. To prepare the input matrix for the group-wise DLSC framework, whole-brain rs-fMRI signals from all voxels for each individual were extracted. After normalization with a mean of zero and a standard deviation of one, the rs-fMRI signals were reorganized into a single data matrix $S_{x}\in {\mathbb{R}}^{t\times n}$ (**Figure 2a**), where *n* refers to the number of rs-fMRI voxels within the mask, and *t* represents the number of time points. The multiple signal matrices for all individuals in both the control and ASD groups were temporally concatenated into a larger matrix $S\in {\mathbb{R}}^{Nt\times n}$, where *N*( N=p+q, p is the number of individuals in the ASD group, and q is the number of individuals in the TDC group) is the total number of individuals aggregated along the time dimension (**Figure 2b**). Note that *S* is composed of two groups of individuals, GT represents the development control group, and GA represents the autism spectrum disorder group:

(1) $$\begin{array}{c} S={\left[S_{G_{T}},S_{G_{A}}\right]}^{T},S_{G_{T}}=={\left[S_{T_{1}},S_{T_{2}},\ldots ,S_{T_{p}}\right]}^{T},S_{G_{A}}=={\left[S_{A_{1}},S_{A_{2}},\ldots ,S_{A_{q}}\right]}^{T} \end{array}$$

Taking *S* as an input, we conducted the online DLSC analysis (32, 54, 55). Signal matrix S was factorized into a signal dictionary matrix *D* and a spatial coefficient matrix *A* (**Figure 2c**). DLSC was then formulated as Equation 2, where $A=[\alpha_{1},\alpha_{1},\ldots \alpha_{n}]$ and ϵ is the reconstruction error. Here, we had two constraints when representing *S* as the product of the dictionary matrix *D* and coefficient matrix *A*: (1) minimizing the reconstruction error (Equation 1), and (2) learning an efficient dictionary and enforcing a sparse constraint on the matrix *A*. Thus, the empirical cost function is summarized in Equation 3 by considering the average loss of representation of *n* signals.

(2) $$\begin{array}{c} S=DA+\epsilon \end{array}$$

(3) $$\begin{array}{c} f_{n}(D)≜\frac{1}{n}\sum_{i=1}^{n}l(s_{i},D) \end{array}$$

where $l(s_{i},D)$ is defined in Equation 4, and λ is an $l_{1}$ regularization parameter used to balance the optimization of the reconstruction error and the sparse constraint.

(4) $$\begin{array}{c} l(s_{i},D)≜\underset{\alpha_{i}\in {\mathbb{R}}^{m}}{\min}\frac{1}{2}∥s_{i}-DA_{i}{∥}_{2}^{2}+\lambda ∥A_{i}{∥}_{1} \end{array}$$

The formulated matrix factorization problem was efficiently solved using the online dictionary learning method (54). Here, we adopted the same assumption used in previous studies (32, 55–57) when assembling the signal dictionary to represent the original rs-fMRI BOLD signals.

The dictionary size (m = 80) and the sparsity regularization parameter (λ = 0.05) were determined through a grid search evaluating m∈{60, 80, 100} and λ∈{0.01, 0.05, 0.10, 0.20, 0.50}. For each parameter combination, the DLSC decomposition was performed on the full temporally concatenated dataset, and the resulting network components were evaluated based on three criteria: (a) reconstruction error, quantified as the normalized Frobenius norm of the residual ( $||S-DA|{|}_{F}^{2}/||S|{|}_{F}^{2}$); (b) the proportion of components rated as neuroanatomically interpretable by two independent raters (defined as components whose spatial peak resided within gray matter and whose spatial extent conformed to known functional neuroanatomical boundaries); and (c) the LOOCV classification accuracy using connectivity features derived from the DLSC-based ROI atlas. The combination of m = 80 and λ = 0.05 achieved the optimal trade-off between reconstruction fidelity and component interpretability while yielding the highest classification accuracy. A detailed summary of the parameter search results is provided in **Supplementary Tables 2** and **3**.

### Quantitative validation of DLSC-derived networks against a canonical functional atlas

To quantitatively evaluate the spatial correspondence between the 80 DLSC-derived functional networks and established resting-state network templates, we computed spatial overlap matrices against the seven-network parcellation developed by Yeo et al. (2011) (26), which was derived from 1,000 neurotypical young adults and is widely adopted as a reference standard in the field. For each DLSC component, a binary mask was generated by thresholding the spatial map at z > 2.0 (corresponding approximately to *p* < 0.05, two-tailed). The Dice similarity coefficient (DSC) was calculated between each DLSC component mask and each Yeo network mask:


$$DSC=\frac{2\times |V_{DLSC}\cap V_{Yeo}|}{|V_{DLSC}|\cup |V_{Yeo}|}$$

In addition, to avoid sensitivity to arbitrary thresholding, Pearson’s spatial correlation coefficient was computed between the unthresholded z-score map of each DLSC component and the probabilistic map of each Yeo network. Each DLSC component was assigned to the Yeo network with which it achieved the highest DSC. The statistical significance of each best-match assignment was assessed using spatial permutation testing. Specifically, the DLSC spatial map was randomly rotated (10,000 permutations) to generate a null distribution of maximum DSC values, and an observed DSC exceeding the 95th percentile of this null distribution was considered significant (*p* < 0.05, family-wise error corrected across the seven Yeo networks). To assess overall concordance, we reported the mean DSC across all 80 components, the number and proportion of components significantly matched to each canonical network, and the distribution of DSC values for each Yeo network.

### Detection of functional connectivity-based biomarkers for ASD

After performing the group-wise DLSC analysis, 80 common functional brain networks across the two groups were generated based on previous ICA and sparse coding studies (27, 32). Then, a set of ROIs was extracted automatically by labeling each region based on the presence of unique features using the Nilearn toolbox (33). Thus, the extracted ROIs served as a data-driven node system, based on which different functional connectivity measures could be derived. To summarize, the analytic pipeline consisted of two distinct stages. In the first stage (network decomposition), the group-wise DLSC approach was applied to the concatenated rs-fMRI data to derive a common set of 80 functional brain networks, from which a set of ROIs was extracted as a data-driven node system. This stage was conducted only once, and the resulting ROIs were held constant for all participants. In the second stage (connectivity-based analysis), functional connectivity measures were computed between all pairs of these fixed ROIs. The resulting connectivity edges underwent feature selection to identify the most discriminative connections, and the selected edges were used for SVM-based classification and two-sample *t*-tests to identify candidate biomarkers. Thus, feature selection operated on the connectivity edges (the links between nodes) rather than on the nodes (ROIs) or networks themselves.

Three complementary functional connectivity measures were computed for each participant to comprehensively characterize interregional interactions among the DLSC-derived ROIs: Pearson’s correlation, partial correlation, and tangent embedding parameterization (33, 58). These measures captured distinct aspects of functional coupling and provided non-redundant information for subsequent classification and biomarker identification, as detailed below.

Pearson’s correlation quantifies the zero-order linear association between the mean BOLD time series of two ROIs. It reflects the total functional coupling between regions, encompassing both direct neural interactions and indirect effects mediated through other brain regions or shared physiological noise sources. Although widely used and intuitively interpretable, correlation-based connectivity may overestimate true functional relationships because of these confounding indirect pathways.

Partial correlation analysis addresses this limitation by estimating the direct linear relationship between two ROIs after regressing out the effects of all other ROIs in the parcellation. Computed from the inverse of the covariance matrix (the precision matrix), partial correlation isolates the unique, conditionally independent association between each ROI pair, thereby reducing the influence of mediating variables and common global signals. However, accurate estimation of the precision matrix requires the number of observations (time points) to substantially exceed the number of variables (ROIs), which can be challenging in high-dimensional parcellations. Accordingly, in the present study, the dimensionality was therefore reduced through the DLSC-based ROI extraction and feature selection steps to ensure stable estimation.

Tangent embedding parameterizes the functional connectivity matrix onto the tangent space of the manifold of symmetric positive definite (SPD) covariance matrices. In this framework, individual covariance matrices are first projected onto a common reference point (typically the group-averaged covariance matrix) using the affine-invariant Riemannian metric and then mapped into the Euclidean tangent space via the matrix logarithm. This transformation yields vectorized connectivity features that respect the geometric structure of covariance matrices, account for inter-subject variability in overall signal scaling, and are particularly well suited for linear classifiers such as support vector machines. Unlike correlation and partial correlation, which are computed directly in the original data space, tangent embedding leverages the Riemannian geometry of the connectivity space to enhance discriminative power and has been demonstrated to achieve superior classification performance in resting-state fMRI-based population studies.

In summary, the three measures provided complementary perspectives on functional brain organization: correlation captured total (direct plus indirect) coupling, partial correlation isolated direct connectivity, and tangent embedding provided a geometrically regularized representation optimized for linear classification. Their simultaneous use allowed us to evaluate the consistency of identified biomarkers across different connectivity definitions and to determine which connectivity formulation yielded the most discriminative information for distinguishing participants with ASD from TDC participants. For each participant, the three functional connectivity matrices were computed and vectorized, yielding a set of connectivity features for all ROI pairs. These feature vectors subsequently served as inputs to the classification and statistical analyses described below.

To validate the effectiveness of the DLSC method in learning the brain ROI atlas, three *l_2_*-penalized linear SVM classifiers were trained using three different connectivity measures to discriminate participants with ASD from the matched TDCs. In addition, leave-one-out cross-validation (LOOCV) (59) was adopted, given the limited sample size, to maximize the use of available data for model training and evaluation. To further assess the generalizability of the classifiers to unseen data, we additionally performed 10-fold random-split cross-validation. Specifically, the entire dataset was randomly partitioned into 10 equal-sized folds, with each fold serving once as the test set while the remaining 9 folds were used for training. This procedure was repeated 20 times with different random seeds to mitigate partition bias, and the mean accuracy and standard deviation across all repetitions were reported (60).

### Multiple comparisons correction

For the between-group comparison of functional connectivity, two-sample *t*-tests (two-tailed) were conducted on each connectivity edge retained after feature selection within each connectivity matrix. The total number of tested edges per matrix was approximately 2,784 (correlation), 2,748 (partial correlation), and 2,740 (tangent embedding), varying slightly across cross-validation folds due to the nested feature selection design. The Benjamini–Hochberg false discovery rate (FDR) procedure (61) was applied to correct for multiple comparisons across all tested edges within each matrix independently, with the FDR threshold set at *q* < 0.05. Connectivity edges with FDR-corrected *p* < 0.05 in a given matrix were considered statistically significant within that matrix.

To reduce error variance and increase statistical power, Pearson’s partial correlation analyses were performed to examine relationships between the identified atypical functional connections and clinical measures, including ADOS communication, ADOS social interaction, CARS, GEM-PR total, GEM-PR cognitive, and GEM-PR affective scores, with age, sex, and IQ included as covariates. The Benjamini–Hochberg FDR procedure was applied to all connection–clinical variable pairs within each connectivity matrix (16 connections × 6 clinical variables = 96 tests per matrix; *q* < 0.05). The FDR-corrected significance levels are reported in the corresponding tables.

It should be acknowledged that the three connectivity measures (correlation, partial correlation, and tangent embedding) were derived from the same underlying BOLD time series and were therefore not statistically independent. Performing FDR correction separately within each matrix may yield a slightly anti-conservative overall false discovery rate if the test statistics are positively correlated across matrices. We adopted per-matrix FDR correction for the following reasons. First, the three connectivity measures served complementary rather than redundant roles in this study: they captured distinct mathematical relationships among BOLD time series (total coupling, conditional independence, and Riemannian tangent space parameterization, respectively), and per-matrix correction preserved the ability to evaluate convergent validity, i.e., which atypical connections were robustly observed across multiple connectivity definitions. Second, connections that survived FDR correction in two or more matrices had a substantially lower joint false-positive probability than those significant in only a single matrix, providing an inherent constraint on spurious findings. Third, this per-matrix reporting approach is consistent with established practices in the rs-fMRI connectomics and biomarker discovery literature. Nevertheless, readers should be aware that the set of declared significant findings across all three matrices may carry an overall false discovery rate that modestly exceeds the nominal per-matrix level, and cross-matrix convergence should be considered supporting rather than confirmatory evidence.

## Results

### Demographics

The ASD group comprised a total of 69 male participants (69/80, 86.250%) and 11 female participants (11/80, 13.750%). The average age of this group was 9.503 ± 2.512 years, and the participants’ average IQ was 103.325 ± 20.968. The TDC group included a total of 37 male participants (37/50, 74%) and 13 female participants (13/50, 26%). The average age of this group was 10.022 ± 3.124 years, and the participants’ average IQ was 108.860 ± 17.660. There were no significant between-group differences in age (*t* = –1.041, *p* = 0.300), sex (χ^2^ = 3.067, *p* = 0.080), or IQ (*t* = –1.553, *p* = 0.123). The mean scores of the three dimensions of ADI-R and ADOS-2 all reached the diagnostic criteria (see **Table 1**).

### Between-group differences in empathy capability

Comparative analysis of the GEM-PR total scores and the cognitive and affective subscales in children and adolescents demonstrated statistically significant differences between the two groups: [ASD group (10.963 ± 18.025) < TDC group (30.020 ± 19.719), *p* = 0.000] for GEM-PR total scores, [ASD group (3.050 ± 10.771) < TDC group (24.540 ± 17.655), *p* = 0.000] for the subscale of cognitive empathy, and [ASD group (12.038 ± 16.282) < TDC group (17.840 ± 13.196), *p* = 0.036] for the affective subscales. Details are presented in **Table 1**.

### Common networks from temporally concatenated sparse coding and validation results

In **Figure 2c**, we set the dictionary size to 80, which was based on previous ICA and sparse coding studies (27, 32). A total of 80 common networks were successfully learned and are displayed in **Figure 1**. These networks included the visual, auditory, motor, executive control, and default mode networks. They encompassed both cortical and subcortical regions, such as the hippocampus, thalamus, and amygdala.

To validate the effectiveness of the DLSC method in the learning brain ROI atlas, we trained three $l_{2}$-penalized linear SVM classifiers by using three different connectivity measures between all pairs of regions as features to classify participants with ASD from the matched TDC participants. To accelerate computation and reduce the influence of non-discriminative connectivity edges, feature selection was embedded within each cross-validation fold to prevent data leakage. Specifically, in each iteration of the leave-one-out cross-validation (LOOCV), the following nested procedure was implemented. The test participant was held out, and SelectFromModel (62), a meta-transformer that selects features based on the absolute coefficients of a linear SVM estimator, was applied exclusively to the training set (comprising the remaining N – 1 participants) to identify the most discriminative connectivity edges. A linear SVM classifier was then trained on the selected feature subset and evaluated on the held-out test participant. This process was repeated so that feature selection was performed independently within each cross-validation fold, ensuring that information from the test set never influenced feature selection. The same nested strategy was applied to the 10-fold random-split cross-validation. Because the set of retained features varied slightly across folds depending on the specific composition of the training set, the feature counts reported in the Results section represent representative values from a single exemplary fold rather than a fixed feature set applied uniformly across all folds. In one representative cross-validation fold, SelectFromModel retained approximately 2,784 connectivity features for the correlation matrix, 2,748 for the partial correlation matrix, and 2,740 for the tangent embedding matrix. The exact number of retained features varied modestly across folds owing to differences in training set composition, consistent with the nested cross-validation design.

Quantitative comparison with the seven-network atlas of Yeo et al. (2011) confirmed the strong spatial correspondence of the DLSC-derived networks with canonical resting-state networks. Across all 80 DLSC components, the mean Dice similarity coefficient with the best-matching Yeo network was 0.52 ± 0.14 (range: 0.21–0.78). Of the 80 components, 62 (77.5%) achieved statistically significant spatial correspondence with one of the seven Yeo networks after spatial permutation testing (*p* < 0.05, corrected). The default mode, visual, and somatomotor networks showed the highest concordance (mean DSCs: 0.61, 0.58, and 0.55, respectively). Notably, several DLSC components exhibited partial overlap with multiple Yeo networks or did not reach significant correspondence with any single canonical network, reflecting finer-grained parcellations or cohort-specific functional organization not fully captured by the neurotypical adult-derived Yeo template. A detailed matrix of DSC values for all 80 components against all seven Yeo networks is provided in **Supplementary Table 1**.

### Significantly altered connections in ASD

**Figure 3:**
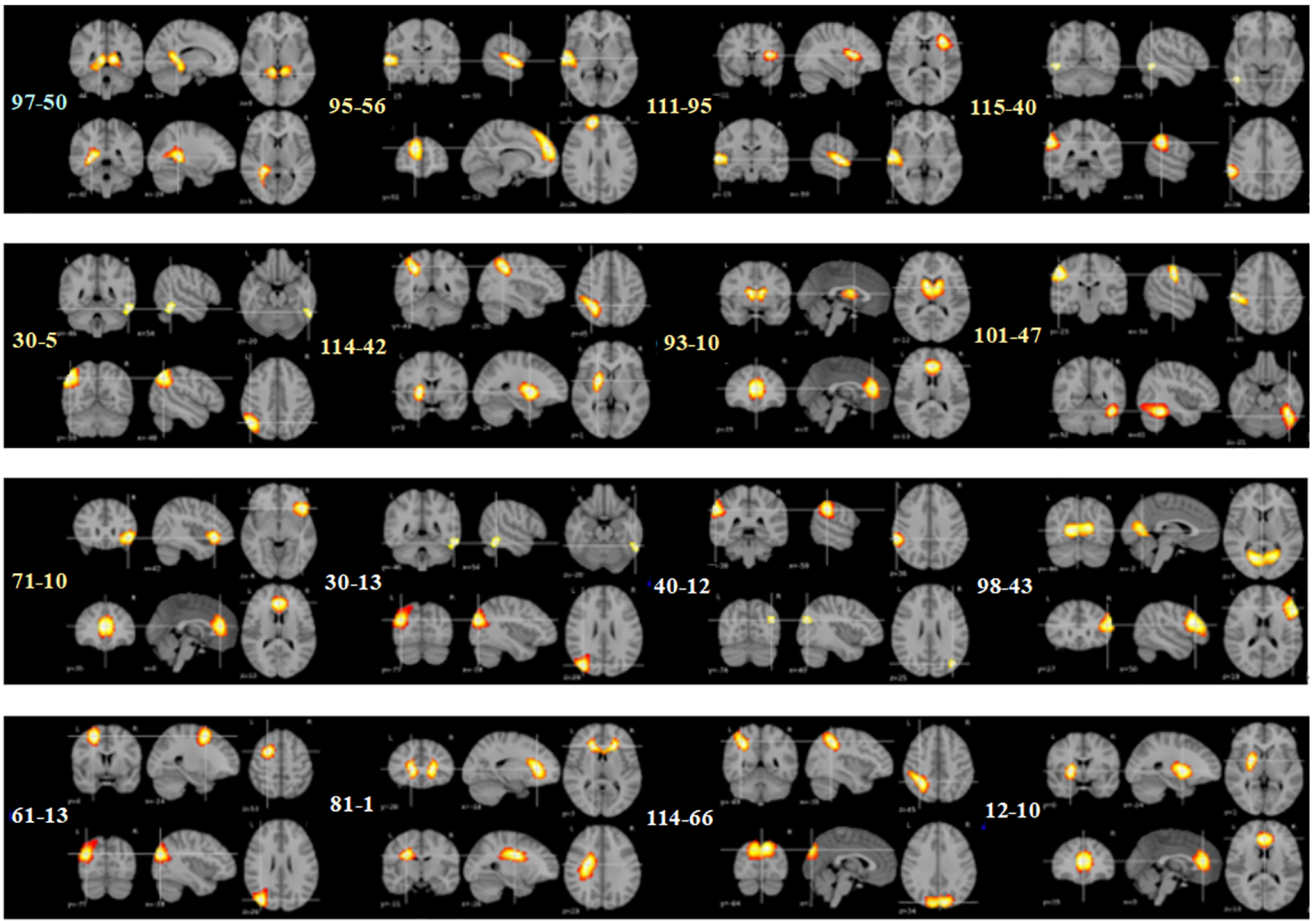
Connectivity biomarkers. Node-pair labels (index numbers of regions of interest shown) are displayed on the left side of each subpanel. The light blue label indicates that, across all three types of connectivity measures, the autism spectrum disorder (ASD) group showed significantly higher values than the typical developmental (TDC) group. Yellow and white labels represent connections identified by two and three types of connectivity measures, respectively, showing significantly lower functional connectivity strengths in the ASD group compared with the TDC group.

To characterize atypical functional connections in ASD, both left- and right-tailed *t*-tests were performed. In total, we identified 16 significantly (*p* < 0.001) altered functional connections, which are shown in **Figure 3** and **Table 2**. Here, *p*-values were corrected for multiple comparisons using the FDR. Further analysis of the functional connectivity results showed that one functional connection in the ASD group was significantly stronger than that in the TDC group (increased; top left in **Figure 3**), whereas the other 15 functional connections were significantly weaker than those in the TDC group (decreased). Among the 15 decreased connections, the connectivity that connected the ROI-115 (left inferior temporal lobe) and ROI-40 (left parietal lobe) belonged to the TPJ, and that which connected ROI-71 (insula) and ROI-10 (anterior cingulate) was located within the salience network (SN). In addition to TPJ connectivity, the other 14 decreased functional connections occurred between different networks derived from the DLSC region, such as the ventral attention network, frontoparietal network, central executive network, limbic system, SN, TPJ, visual system, and DMN (26).

**Table 2:** The brain regions (regions of interest) shown in **Figure 3**.

Label	Brain region – brain region
97–50	Parahippocampal gyrus/Lingual – Temporal_Inf_L/hippocampus_L
95–56	Temporal_Mid_L/Temporal_Sup_L **–** Frontal_Mid_L/Frontal_Sup_L
111–95	Insula_R/Putamen_R – Temporal_Sup_L/Temporal_Mid_L
115–40	Temporal_Inf_L – Parietal_Inf_L/SupraMarginal_L
30–5	Temporal_Inf_R – Angular_L
114–42	Parietal_Inf_L – Putamen_L/Amygdala_L
93–10	Thalamus/Caudate – Cingulum_Ant
101–47	Postcentral_L – Fusiform_R
71–10	Insula_R – Cingulum_Ant
30–13	Temporal_Inf_R – Occipital_Mid_L
40–12	SupraMarginal_L – Occipital_Mid_R
98–43	Calcarine – Frontal_Mid_R/frontal_Inf_Tri_R/Frontal_Inf_Orb_R
61–13	Frontal_Mid_L – Occipital_Mid_L
81–1	Caudate – Insula_L
114–66	Parietal_Inf_L – Occipital_Sup
42–10	Putamen_L/Amygdala_L – Cingulum_Ant

The brain regions shown in **Figure 3** are described in **Table 2** using the Automated Anatomical Labeling template. L, left; R, right; Ant, anterior; Mid, middle; Inf, inferior; Tri, triangle; Orb, orbitofrontal. The red label indicates that, across all three types of connectivity measures, the autism spectrum developmental (ASD) group showed significantly higher values than that the typical developmental (TDC) group. Light blue and deep blue labels represent connections identified by two and three types of connectivity measures, respectively, showing significantly lower functional connectivity strengths in the ASD group compared with the TDC group.

### Relationship between brain functional connections and clinical measures

To reduce error variance and increase statistical power, we conducted a partial correlation analysis using age, sex, and IQ as covariates between the atypical functional connections and the empathy abilities. The Benjamini–Hochberg false discovery rate (FDR) correction was applied to the correlation analyses within each connectivity matrix separately to control for multiple comparisons (**Tables 3**–**5**). It should be noted that full-scale IQ (WISC-CR) was not included as a correlate in the brain–behavior association analyses. This decision was based on the following considerations: ① restricted within-group variance of IQ in this specific sample and insufficient statistical power; ② lack of *a priori* functional correspondence between the identified functional connections and general intelligence; and ③ avoidance of further increases in the multiple comparison burden and dilution of the analytical focus. The results showed that there were positive correlations between some functional connections and GEM scores, including the following: 111 (right insula/right putamen) − 95 (left superior temporal/left middle temporal) and GEM-affective score (r = 0.249); 40 (left supra marginal) − 12 (right middle occipital) and GEM-affective score (r = 0.241); 114 (left inferior parietal) − 66 (superior occipital) and GEM-affective score (r =0.232); 42 (left putamen/left amygdala) − 10 (anterior cingulum) and GEM-affective score (r =0.297); 93 (thalamus/caudate) − 10 (anterior cingulum) and GEM-affective score (r = 0.240); and 81 (caudate) – 1 (left insula) and GEM-total score (r = 0.236). We also found negative correlations between some functional connections and other clinical scores as follows: 30 (right inferior temporal) – 13 (left middle occipital) and ADOS-communication score (r = −0.285)], and 81 (Caudate) − 1 (left Insula) and CARS score (r = −0.262).

**Table 3:** Correlation analysis of atypical functional connections and clinical features in the correlation matrix.

Variables	ADOS-2		CARS	Total	GEM-PR	Affective
	Communication	Social interaction			Cognitive	
97–50	-0.099	-0.006	0.204	0.070	0.159	0.038
95–56	-0.107	0.002	-0.110	-0.96	-0.021	-0.026
111–95	-0.115	-0.145	-0.198	0.160	0.115	0.249*
30–13	-0.285*	-0.202	-0.149	-0.001	-0.094	0.107
40–12	-0.076	-0.025	-0.205	0.146	0.097	0.241*
98–43	-0.076	-0.029	-0.210	0.058	0.015	0.168
61–13	-0.136	-0.113	-0.013	0.185	0.191	0.209
81–1	-0.202	-0.138	-0.262*	0.236*	0.218	0.306**
114–66	-0.073	-0.071	-0.031	0.144	0.088	0.232*
42–10	-0.236*	-0.110	-0.177	0.207	0.193	0.297*
93–10	-0.162	-0.080	-0.188	0.201	0.212	0.24*

ADOS-2, Autism Diagnostic Observation Schedule, Second Edition; CARS, Childhood Autism Rating Scale; and GEM-PR, Griffith Empathy Measure-Parent Ratings. The labels of the brain regions are shown in **Table 2**. ^*^p < 0.05, ^**^p < 0.01. Bold values indicate statistical significance.

**Table 4:** Correlation analysis of atypical functional connections and clinical features in the partial matrix.

Variables	ADOS-2		CARS	Total	GEM	Affective
	Communication	Social interaction			Cognitive	
97–50	0.072	-0.092	0.051	-0.162	-0.670	-0.164
95–56	0.011	-0.104	-0.081	-0.257*	-0.237*	-0.244*
111–95	0.076	-0.087	0.179	-0.016	0.009	-0.065
115–40	0.020	0.041	0.039	-0.006	0.019	-0.034
30–5	-0.173	0.021	0.027	0.055	0.091	0.084
114–42	-0.181	-0.125	-0.039	-0.124	-0.114	-0.065
93–10	-0.039	-0.017	-0.114	-0.016	0.041	-0.042
101–47	-0.104	-0.104	-0.117	0.170	0.128	0.155
71–10	-0.179	0.100	-0.208	0.163	0.257*	0.090
30–13	-0.110	-0.102	0.160	-0.103	-0.190	-0.036
40–12	-0.063	-0.115	-0.087	-0.082	-0.153	-0.018
98–43	0.075	-0.030	0.1112	-0.084	-0.208	-0.007
61–13	-0.022	0.049	0.133	-0.068	-0.082	-0.073
81–1	-0.283*	-0.004	-0.148	0.030	0.121	0.009
114–66	0.072	-0.123	0.064	0.028	0.035	-0.028
42–10	-0.129	-0.140	-0.02	0.177	0.105	0.205

ADOS-2, Autism Diagnostic Observation Schedule, Second Edition; CARS, Childhood Autism Rating Scale; and GEM-PR, Griffith Empathy Measure-Parent Ratings. The labels of the brain regions are shown in **Table 3**. ^*^p < 0.05, ^**^p < 0.01. Bold values indicate statistical significance.

**Table 5:** Correlation analysis of atypical functional connections and clinical features in the tangent matrix.

Variables	ADOS-2		CARS	Total	GEM	Affective
	Communication	Social interaction			Cognitive	
97–50	0.058	-0.023	0.142	-0.109	0.003	-0.132
115–40	0.000	-0.032	0.014	0.0810	0.092	0.064
30–5	-0.111	0.031	-0.026	0.004	0.074	0.021
114–42	-0.249*	-0.170	-0.088	-0.100	-0.078	-0.062
101–47	-0.062	-0.136	-0.109	0.170	0.110	0.169
71–10	-0.156	0.124	-0.289*	0.178	0.266*	0.106
30–13	-0.159	-0.153	0.114	-0.130	-0.244*	-0.037
40–12	-0.070	-0.137	-0.158	-0.021	-0.073	0.048
98–43	0.093	0.029	-0.025	-0.085	-0.181	-0.006
61–13	-0.070	0.030	0.082	0.035	0.042	0.007
81–1	-0.265*	-0.061	-0.196	0.017	0.074	0.032
114–66	0.043	-0.153	0.136	0.116	0.057	0.113
42–10	-0.231*	-0.099	-0.007	0.191	0.136	0.234*
93–10	-0.126	-0.033	-0.166	-0.016	0.056	-0.042

ADOS-2, Autism Diagnostic Observation Schedule, Second Edition; CARS, Childhood Autism Rating Scale; and GEM-PR, Griffith Empathy Measure-Parent Ratings. The labels of the brain regions are shown in **Table 3**. ^*^p < 0.05, ^**^p < 0.01. Bold values indicate statistical significance.

#### a) Partial correlations

Based on the partial correlation matrix, a positive correlation was observed between the functional connection of 71 (right insula) – 0 (anterior cingulum) and GEM-cognitive score (r = 0.257). Negative correlations were observed between 81(caudate) − 1 (left insula) and ADOS-2-communication score (r = −0.283), and between 95 (left superior temporal/left middle temporal) − 56 (left superior frontal/left middle frontal) and GEM-total score (r = −0.257), GEM-cognitive score (r = −0.237), and GEM-affective score (r = −0.244).

#### b) Tangent embedding

Based on tangent embedding parameterization of the covariance matrix, positive correlations were observed between the functional connection of 71 (right insula) − 10 (anterior cingulum), 42 (left putamen/left amygdala) − 10 (anterior cingulum) and GEM-cognitive score (r_71-10_ = 0.266, r_42-10_ = 0.234). Negative correlations were observed between the functional connection of 30 (right inferior temporal) – 13 (left middle occipital) and GEM-cognitive score (r_30-13_ = −0.244), 114 (left inferior parietal) – 42 (left putamen/left amygdala), 81 (caudate) − 1(left insula), 42 (left putamen/left amygdala) − 10 (anterior cingulum), and ADOS-2-communication score (r_114-42_ = −0.249, r_81-1_ = −0.265, r_71-10_ =, r_42-10_ = −0.231), and 71 (right insula) − 10 (anterior cingulum) and CARS (r = −0.289).

## Discussion

### Classification accuracy

In this study, we used a novel group-wise sparse representation method to analyze resting-state fMRI data from individuals with ASD. The advantages of this method are summarized below. First, the group-wise common dictionary bases were learned and optimized from the whole-brain rs-fMRI data across all cohorts. They were therefore richer than traditional methods in assessing connectome-scale information encoded in the entire rs-fMRI data. Second, a commonly learned dictionary effectively exploited both the similarities and differences between individuals and groups, making the matrix more robust to noise and more powerful in detecting cross-group differences, an advantage that is valuable when combined with systematic clinical evaluations. Finally, compared with previous sparse representations of rs-fMRI signals of individual brains for network analysis (53, 56), our group-wise statistical method automatically established correspondence between different individuals and systematically evaluated differences in functional activities among these individuals. We established the correspondence relationship of each component network by learning the basic dictionary foundation from different groups and individuals, and guided the spatial normalization and signal extraction of the single brain through the common mask, thus providing the basis for statistical analyses and comparisons between groups. In our dataset, this approach classified participants with ASD and control subjects with an accuracy of 95% (correlation matrix), 100% (partial correlation matrix), and 100% (tangent embedding matrix). By comparing the between-group differences based on the functional brain connectivity established by the common dictionary, our classification results showed that using DLSC extracted effective and robust brain ROI atlases.

Quantitative spatial overlap analysis confirmed the strong convergence between the DLSC-derived networks and the Yeo et al. (2011) canonical networks. Overall, 77.5% of DLSC components achieved significant spatial correspondence with one of the seven Yeo networks (mean DSC = 0.52, spatial permutation test, *p* < 0.05 corrected). The highest concordance was observed for the default mode, visual, and somatomotor networks (mean DSCs: 0.61, 0.58, and 0.55, respectively). However, the remaining 22.5% of DLSC components did not exhibit significant one-to-one correspondence with any single Yeo network. This pattern likely reflects the more granular parcellation afforded by the sparsity constraint (e.g., the fractionation of the salience network into dorsal and ventral insular components), in addition to potential cohort-specific functional architecture in this pediatric ASD sample that may not be fully captured by the neurotypical adult-derived template.

### Alterations in empathy-related functions and their clinical implications

Empathy, which is the capacity to recognize, understand, and share the emotional states of others, is a multidimensional construct encompassing both cognitive components (the ability to adopt and reason about another person’s mental perspective, often termed “theory of mind” or “mentalizing”) and affective components (the capacity to experience an appropriate emotional response to another person’s affective state) (4, 5). Empathic ability is fundamental to successful social interaction, relationship formation, and prosocial behavior, and its impairment is widely recognized as a core feature of ASD that contributes substantially to the social communication difficulties characteristic of the disorder (2, 3). Understanding the neural substrates of empathy deficits in ASD is therefore of basic neuroscientific interest and has direct clinical implications. Identifying the specific neural circuits underlying impaired empathy may inform the development of targeted interventions, including social skills training, cognitive-behavioral approaches, and neuromodulation techniques, that aim to improve social functioning and quality of life for individuals with ASD. Moreover, establishing reliable neural correlates of empathy deficits may provide objective biomarkers for monitoring treatment responses and for stratifying patients in clinical trials, addressing a critical unmet need given the current reliance on behavioral assessments that can be influenced by factors such as rater bias, participant motivation, and developmental level.

In the present study, we found that children and adolescents with ASD exhibited significantly lower total GEM-PR scores and lower scores on both the cognitive and affective empathy subscales, compared to age-, sex-, and IQ-matched typically developing controls. These findings are broadly consistent with a substantial body of previous research documenting empathy impairments in ASD. Using the Griffith Empathy Measure in a sample of 57 individuals aged 9–24 years, Deschamps et al. (63) similarly reported that school-age children with ASD had lower cognitive and affective empathy scores than typically developing peers. Studies employing the Empathy Quotient (EQ) have also demonstrated significantly reduced empathy in ASD across wide age ranges, from preschool children to older adults (64, 65), indicating that empathy deficits are a pervasive and developmentally stable feature of the disorder. Our results thus replicate and extend these previous findings within a well-characterized sample of children and adolescents with ASD using a validated parent-report measure with established reliability in the Chinese population (35).

A key contribution of our study was the demonstration that individual differences in empathy among individuals with ASD were associated with specific patterns of resting-state functional connectivity within and between the salience network, the temporoparietal junction, and the broader social brain network. These findings bridge the behavioral and neural levels of analysis and provide a mechanistic framework for understanding the neurobiological underpinnings of empathy heterogeneity in ASD. On the cognitive empathy dimension, our finding of a positive correlation between right insula–ACC connectivity (the core SN pathway) and GEM-PR cognitive scores is consistent with a well-established literature implicating the SN and the ToM network in mental abilities (66–69). The anterior insula and ACC are thought to support cognitive empathy through their role in integrating interoceptive information about one’s own emotional state with external cues regarding the mental states of others, a process that enables the simulation-based inference that underlies perspective-taking and mental state attribution (70–72). Our results are consistent with this model and suggest that reduced functional coupling within the SN may impair this integrative process, contributing to the characteristic mentalizing deficits observed in ASD.

On the affective empathy dimension, we found that connectivity between the left amygdala and both the anterior cingulum (connection 42–10) and the left inferior parietal region (connection 114–42), together with connectivity between the fusiform gyrus and the postcentral gyrus (connection 101–47), was associated with GEM-PR affective scores. These findings are consistent with the established roles of the amygdala in emotional reactivity and salience detection and of the fusiform gyrus in face and identity processing (73–75). However, the literature on affective empathy in ASD is less consistent than that on cognitive empathy. Some studies have reported reduced emotional contagion and affective sharing in ASD, while others have found intact or even heightened affective responsiveness, particularly in response to distress cues (63, 76). Our finding of reduced connectivity within emotion-related circuits, coupled with lower parent-reported affective empathy, supports the former interpretation. However, we note that self-report and behavioral measures of affective empathy may yield different patterns of results than parent-report measures. Future studies incorporating multi-method assessment of both cognitive and affective empathy, including self-report, parent-report, and behavioral or physiological indices, will be necessary to fully characterize the relationship between neural connectivity and the distinct components of empathic function in ASD.

Our findings contribute to a growing body of evidence indicating that empathy deficits in ASD are not monolithic but are differentially associated with specific neural circuits. The cognitive dimension of empathy appears most closely tied to the functional integrity of the SN and ToM networks, while the affective dimension additionally involves limbic and ventral temporal regions supporting emotion processing and social perception. This dissociation has potential clinical implications. It suggests that targeted interventions aimed at enhancing mentalizing abilities (cognitive empathy) may benefit from strategies that strengthen SN-mediated interoceptive awareness and perspective-taking, whereas interventions aimed at enhancing affective empathy may need to additionally target amygdala-mediated emotional reactivity and social reward processing. Furthermore, the identification of specific functional connectivity patterns associated with distinct empathy dimensions raises the possibility of developing individually tailored intervention approaches guided by each patient’s unique neural connectivity profile.

### Atypical functional connectivity patterns and their functional significance in ASD

The between-group comparison identified 16 functional connections that were significantly altered in the ASD group relative to the TDC group, with the majority (15 of 16) exhibiting reduced connectivity. This predominance of hypoconnectivity is consistent with the widely reported pattern of reduced functional integration in ASD documented across multiple independent resting-state fMRI studies with moderate to large samples, including those from the ABIDE consortium (77–79). Rather than discussing each connection in isolation, we organized the following discussion around the core functional networks implicated by these atypical connections and interpreted their potential contributions to the social, cognitive, and affective features of ASD.

① Salience Network (SN). Several of the identified atypical connections involved core nodes of the SN, most notably the right insula (node 71) and the anterior cingulate cortex (ACC; node 10). The reduced connectivity between these regions, which is a canonical SN pathway, was observed across multiple connectivity measures and showed positive correlations with cognitive empathy scores. The SN is critically involved in detecting behaviorally relevant stimuli, integrating interoceptive and exteroceptive information, and facilitating dynamic switching between the default mode and central executive networks (80, 81). In typically developing individuals, the anterior insula and ACC jointly support the embodied simulation of the emotional and mental states of others, a process that is fundamental to both affective and cognitive empathy (70–72). Disrupted insula and ACC coupling has been reported in ASD across multiple independent studies, and has been associated with deficits in social cognition, emotional awareness, and empathy (4, 5, 82). Our findings extend this literature by demonstrating that reduced SN connectivity is specifically correlated with lower empathy scores in children and adolescents with ASD, thereby providing a direct link between SN dysfunction and one of the core behavioral features of the disorder. Beyond the insula-ACC axis, our analysis also identified atypical connectivity between the insula and the caudate (connection 81–1) and between the insula and temporal regions (connection 111–95), suggesting that SN dysfunction in ASD extends to its interactions with subcortical and association networks, potentially affecting reward processing and social perception.

② Temporoparietal junction (TPJ) and the theory of mind (ToM) network. The connection between the left inferior temporal region and the left inferior parietal/supramarginal region corresponds to the temporoparietal junction, a cortical hub of the ToM network. The TPJ is consistently activated during tasks requiring perspective-taking, mental state attribution, and the distinction between self and other representations (8, 83). Reduced connectivity within the TPJ, as observed in our ASD cohort, is consistent with a substantial body of neuroimaging literature documenting atypical TPJ function and connectivity in ASD (68, 69). Notably, the TPJ does not operate in isolation but interacts extensively with other nodes of the social brain network, including the medial prefrontal cortex, the superior temporal sulcus, and the temporal poles (8). The co-occurrence of TPJ hypoconnectivity and alterations in other social brain regions in our study suggests a broader disruption of the distributed circuitry supporting mentalizing and social cognition in ASD.

③ Social brain network (SBN) and emotion processing. Multiple atypical connections in our study involved regions that are core components of the social brain network. The left amygdala (node 42) showed reduced connectivity with both the anterior cingulum (connection 42–10) and the left inferior parietal region (connection 114–42). The amygdala plays a central role in emotion processing, salience detection, and the coordination of autonomic responses to socially relevant stimuli (73). Atypical amygdala connectivity in ASD has been documented in both resting-state and task-based fMRI studies, with some reports describing reduced amygdala-prefrontal coupling during emotion regulation and others describing amygdala hyperreactivity followed by reduced habituation (74, 75). Our finding of reduced amygdala–ACC and amygdala–parietal connectivity extends this literature by demonstrating that these alterations are present at rest and are associated with empathy deficits. Additionally, the fusiform gyrus (node 47), a region specialized for face perception and personal identity recognition, showed atypical connectivity with the postcentral gyrus (connection 101–47), consistent with previous evidence of altered fusiform function and connectivity in ASD during face processing tasks (73).

④ Default mode network (DMN) and self-referential processing. Several connections involving DMN regions, including the left angular gyrus (node 5), the left superior medial frontal cortex (node 56), and the right middle frontal gyrus (node 43), were found to be altered in the ASD group. The DMN is engaged during self-referential thought, autobiographical memory, and social cognition, and atypical DMN connectivity has been consistently associated with ASD across numerous studies (11–14, 84). In our study, the connectivity between the left superior temporal/left middle temporal region and the left superior frontal/left middle frontal region (connection 95–56) was negatively correlated with empathy scores in the partial correlation matrix, suggesting that atypical communication between the DMN and language-related temporal regions may contribute to difficulties in self-referential emotional processing and social communication in ASD.

⑤ Visual and cross-network integration. The inclusion of visual network nodes among the atypical connections, including the calcarine cortex (node 98), the middle occipital gyrus (node 13), and the superior occipital cortex (node 66), is noteworthy. Although visual processing has historically received less attention in ASD social cognition research, accumulating evidence suggests that altered visual-social network interactions may reflect atypical integration of perceptual and social information in ASD (26). The atypical connections between visual regions and frontal, parietal, and subcortical nodes observed in our study may indicate disrupted top-down modulation of visual processing by higher-order association networks, potentially contributing to the reduced attention to socially relevant visual cues (e.g., faces and biological motion) that characterizes ASD.

⑥ Subcortical–cortical circuits. Several atypical connections implicated subcortical structures, including the caudate (node 81), putamen (nodes 42 and 114), and thalamus (node 93), in combination with cortical regions within the SN and SBN. The basal ganglia and thalamus are integral components of cortico-striatal-thalamic loops that support reward processing, habitual behavior, and cognitive flexibility (81). Atypical striatal functional connectivity has been reported in children with ASD and has been associated with restricted and repetitive behaviors (85). In our study, subcortical–cortical connectivity involving the caudate and insula (connection 81–1) was correlated with both empathy scores and ADOS communication scores across multiple connectivity measures, suggesting that these circuits may contribute not only to restricted and repetitive behaviors but also to impairments in social communication in ASD.

In summary, the 16 atypical functional connections identified in this study collectively implicated a distributed set of brain networks involved in salience detection, interoception, mentalizing, emotion processing, self-referential thought, and sensory integration. Rather than reflecting isolated regional dysfunctions, these findings suggest that ASD is characterized by a systems-level disruption of functional integration within and between the salience, social brain, default mode, and subcortical networks, which together support the complex empathic and social cognitive abilities impaired in the disorder. The convergence of our findings with previous resting-state and task-based ASD studies support the validity of these connectivity alterations as candidate biomarkers while also highlighting the need for future research to examine the developmental trajectories of these network-level disruptions and their causal relationships with ASD symptomatology.

### Potential neural mechanisms underlying empathy-related functional connectivity alterations

The observed correlations between specific functional connections and empathy scores in our study provided insights into the neural substrates of empathic dysfunction in ASD, particularly implicating the salience network (SN) and the TPJ. The positive correlation between right insula-anterior cingulate cortex (ACC) connectivity (nodes 71–10, a core SN pathway) and cognitive empathy scores in both the partial correlation and tangent embedding matrices may have reflected the integrative role of the SN in supporting higher-order mentalizing processes. The anterior insula is critically involved in representing visceral and affective bodily states (86), while the ACC facilitates conflict monitoring and salience detection; together (87), these regions enable the embodied simulation that underpins cognitive empathy (88, 89). Reduced functional coupling along this fronto-insular pathway in individuals with ASD may therefore impair the ability to infer the mental states of others, consistent with prior evidence of SN dysfunction in ASD (90, 91).

Importantly, a negative correlation emerged between left superior temporal–left middle frontal connectivity (nodes 95–56) and both cognitive and affective GEM scores in the partial correlation matrix. The left superior temporal region contributes to processing socio-affective auditory and language cues, whereas the left middle frontal gyrus subserves controlled retrieval of social knowledge. The inverse relationship with empathy suggested that heightened connectivity along this frontotemporal axis may reflect inefficient or compensatory processing of social-emotional information rather than effective network specialization, a pattern previously documented in ASD populations (92, 93).

Regarding the TPJ (nodes 115–40), its significantly reduced connectivity in the ASD group aligned with the well-established role of this region in theory of mind and perspective-taking (94, 95). As a critical hub within the social brain network, the TPJ enables the distinction between self and other mental representations. Its functional decoupling from other association cortices may directly contribute to the mentalizing deficits and lower cognitive empathy observed in our ASD cohort. Together, these findings suggest that empathy alterations in ASD arose from disrupted functional integration within and between the SN, the ToM network (including the TPJ hub), and broader social brain networks, affecting both the embodied simulation and higher-order inferential components of empathic processing.

### Sex differences in ASD brain connectivity and empathy: implications for the present findings

A growing body of research has demonstrated that the neurobiological substrates of ASD differ between male and female individuals, challenging the long-standing assumption that findings from male-dominated samples can be straightforwardly generalized to female individuals on the autism spectrum. Although the substantial sex imbalance in the present sample, with male participants comprising 86.25% of the ASD group and 74.00% of the TDC group, reflects the well-established approximately 4:1 male-to-female ratio in ASD prevalence (96), it precluded formal sex-stratified analyses. Nevertheless, it is important to consider how sex differences may influence the interpretation of our findings and to identify key areas for future investigation.

In the domain of brain connectivity, several independent resting-state fMRI studies have reported sex-specific patterns of functional connectivity in ASD. Li et al. (97) found that female individuals with ASD exhibited distinct patterns of intrinsic functional connectivity compared with male individuals with ASD, particularly within the default mode network, salience network, and sensorimotor circuits, with some alterations appearing more pronounced in female participants. Alaerts and colleagues (98) similarly reported that male and female individuals with ASD showed partially divergent patterns of resting-state connectivity, with female participants demonstrating alterations that were not merely attenuated versions of the male-typical pattern but instead involved distinct spatial topographies. A recent large-scale analysis of the ABIDE dataset further confirmed that sex moderates the relationship between ASD diagnosis and functional connectivity across multiple large-scale networks, including those most directly implicated in our findings: the salience network, the default mode network, and regions of the social brain (87). These studies suggest that the connectivity-based biomarkers we identified, particularly those involving the SN (e.g., insula–ACC connectivity) and the TPJ, may reflect male-biased patterns of atypical connectivity. In addition, the functional network architecture underlying ASD in female individuals may differ in important ways that our study did not capture.

Sex differences are also highly relevant to the empathy-related findings of the present study. In the general population, robust sex differences in empathy have been documented. Female individuals consistently score higher than male counterparts on self-report measures of both cognitive and affective empathy (99–101), show greater empathic accuracy in behavioral paradigms (102, 103), and exhibit differential patterns of neural activation during empathy tasks, with female individuals typically engaging social brain regions, including the TPJ, anterior insula, and medial prefrontal cortex, more strongly during empathic processing (104, 105). In individuals with ASD, emerging evidence suggests that sex may modulate the relationship between autistic traits and empathy (106, 107). Some studies have reported that the sex difference in empathy observed in the neurotypical population is attenuated in ASD (108), while others have suggested that female individuals with ASD may employ compensatory cognitive strategies, sometimes termed “camouflaging,” which mask social-cognitive difficulties during certain types of behavioral assessments even though underlying neural differences persist (109, 110). In the context of our study, the male-dominated sample may have influenced both the behavioral empathy profiles and the connectivity-empathy correlations we observed. Specifically, the positive correlations we found between SN connectivity and cognitive empathy, together with those between amygdala-related connectivity and affective empathy, may have predominantly reflected male-typical neural mechanisms of empathic processing. Whether these same neural circuits support empathy in female individuals with ASD, or whether these female individuals recruit additional or alternative networks to compensate for dysfunction in the SN or the ToM network (including the TPJ hub), remains an open question of considerable clinical significance.

The limited number of female participants in our sample (11 in the ASD group and 13 in the TDC group) precluded formal testing of sex-by-diagnosis interactions. However, the literature reviewed above suggests that several important questions merit future investigation: whether the direction and magnitude of atypical functional connectivity differ between male and female individuals with ASD, whether the relationship between functional connectivity and empathy is moderated by sex, and whether the DLSC-derived functional network atlas adequately represents the functional architecture of the female ASD brain. Addressing these questions will require deliberate, multisite recruitment strategies to assemble sufficiently large cohorts of female participants with ASD, a critical priority for the field, as highlighted by recent consensus statements (111, 112). Future studies employing sex-by-diagnosis factorial designs, combined with sex-normed clinical assessments and quantitative measures of pubertal stage and sex hormone levels, will be essential for disentangling the contributions of biological sex, sex-related social experience, and ASD diagnosis to the neural and behavioral phenotypes of empathy.

### Internal validation and considerations for generalizability

An important consideration for any data-driven biomarker discovery approach is whether the identified connectivity features reflect robust group-level differences that are likely to replicate in independent samples. In this study, we employed complementary internal validation strategies to evaluate both the stability of the classification performance and the consistency of the identified atypical connections, while acknowledging that external validation remains an essential next step.

Several aspects of the current study support the internal robustness of the DLSC-based connectivity biomarkers. First, classification performance was evaluated using LOOCV and repeated 10-fold random-split cross-validation (20 independent repetitions with different random seeds), with feature selection nested within each cross-validation fold to prevent data leakage (60). The consistently high classification accuracies observed across both cross-validation paradigms, with the 10-fold cross-validation yielding mean accuracies of 93.4% ± 1.8%, 98.7% ± 0.9%, and 99.1% ± 0.7% for correlation, partial correlation, and tangent embedding, respectively, indicate that the discriminative connectivity features were stable with respect to data partitioning within this sample. The small variance across repeated random splits further suggests that the classification performance was not driven by idiosyncratic properties of a particular train-test partition.

Second, the convergence of findings across the three complementary connectivity measures provided an additional layer of internal corroboration. Several atypical connections, including those involving the salience network (insula-anterior cingulate), the temporoparietal junction, and amygdala-cingulate pathways, were identified as significantly altered in two or all three connectivity metrics. Because correlation, partial correlation, and tangent embedding captured distinct statistical relationships among BOLD time series (total coupling, conditional independence, and Riemannian tangent space parameterization, respectively), the observation that certain connections were consistently identified across these mathematically distinct measures argues against their being artifacts of a particular connectivity estimator and suggests that they reflect genuine alterations in neural circuit function within this sample.

Third, the atypical connections identified in this study converge with findings reported in independent ASD resting-state fMRI studies that employed different parcellation schemes, analytic approaches, and participant cohorts (90, 95, 113). The involvement of the salience network, default mode network, and social brain regions in the pathophysiology of ASD has been documented across multiple sites and laboratories, lending indirect support to the neurobiological plausibility of the present findings.

Despite these internal validation results, several important constraints on generalizability must be explicitly acknowledged. The current sample, while carefully characterized, was relatively modest in size (N = 130), was recruited from a single clinical site, and was demographically homogeneous (predominantly male participants, school-age, Han Chinese, all without intellectual disability). These characteristics may limit the extent to which the specific DLSC-derived network components and the set of discriminative connectivity edges identified here generalize to the broader ASD population, which encompasses individuals across a wide range of ages, cognitive and language profiles, sex identities, and cultural and socioeconomic contexts. The classification accuracies reported here, although high, were obtained through internal cross-validation within this specific sample and should not be interpreted as estimates of performance on an independent, external dataset. Internal cross-validation, even when properly nested, provides an estimate of generalization error within the population from which the sample was drawn, assuming that the sample is representative of that population; however, it does not substitute for external validation in a truly independent cohort.

Furthermore, the stability of the DLSC decomposition with respect to the choice of hyperparameters (m = 80, λ = 0.05) has not been systematically evaluated. Although these values were selected based on prior literature and preliminary experimentation, it is possible that different hyperparameter settings could yield network atlases with somewhat different spatial topologies, which could in turn affect the specific connectivity edges identified as atypical. The sensitivity of the findings to these methodological choices remains an open empirical question.

To move from internal validation to demonstrated generalizability, several steps are necessary. First, external cross-validation, in which the DLSC dictionary is learned on an independent dataset and applied to a held-out dataset for connectivity feature extraction and classification, would provide a more direct test of the generalizability of the learned network atlas and the identified biomarkers. The Autism Brain Imaging Data Exchange (ABIDE) (79), which aggregates resting-state fMRI data from multiple international sites, provides a suitable platform for such external validation, although site-related heterogeneity in acquisition parameters and demographic composition must be carefully addressed (114). Second, systematic hyperparameter sensitivity analyses evaluating the consistency of the DLSC decomposition and the resulting classification performance across a range of dictionary sizes (e.g., m = 60, 80, 100, and 120) and sparsity levels (e.g., λ = 0.01, 0.05, 0.1, and 0.2) would provide valuable information regarding the robustness of the method to parameter variation. Third, replication in demographically diverse cohorts, with deliberate oversampling of female participants and inclusion of participants across a broader range of intellectual functioning and language abilities, would establish the boundaries within which the findings are applicable. We regard these as essential priorities for ongoing and future research. Until such external validation is completed, the connectivity-based biomarkers identified in this study should be regarded as promising but preliminary candidates whose clinical utility remains to be established.

## Limitations

This research had several limitations. First, participant recruitment was challenging due to strict inclusion criteria related to IQ and age, resulting in a small sample size. Second, the substantial sex imbalance in our sample, with male participants accounting for 86.25% in the ASD group and 74.00% in the TDC group, represents an important limitation. Although this male predominance reflects the well-documented higher prevalence of ASD in male individuals (approximately a 4:1 male-to-female ratio in epidemiological studies (96)), it may have introduced systematic biases into our findings in several ways. Growing evidence suggests that the neurobiological substrates of ASD differ between male and female individuals, with female individuals exhibiting distinct patterns of functional connectivity alterations, potentially involving more widespread network disruptions or compensatory mechanisms that differ from those observed in male individuals (115, 116). Moreover, sex differences in empathy-related neural processing have been documented in both neurotypical and ASD populations, with female individuals generally demonstrating greater engagement of social brain regions, including the temporoparietal junction and anterior insula, during empathic tasks (104, 117). Consequently, the male-dominated sample may have biased our identified functional connectivity-based biomarkers toward male-specific ASD pathology, potentially limiting the generalizability of our findings to female individuals with ASD. The limited number of female participants also precluded a sex-stratified analysis that could have revealed sex-specific patterns of atypical connectivity in empathy-related networks, particularly within the salience network and social brain networks. Third, the study relied exclusively on sparse coding and dictionary learning methods, without incorporating other data-driven or atlas-based approaches to validate findings across whole-brain and regional analyses.

Furthermore, the FDR correction for the between-group and brain-behavior correlation analyses was performed separately within each of the three connectivity matrices. Because the three connectivity measures were derived from the same underlying data, this per-matrix correction strategy may be slightly anticonservative; however, the key findings of this study, particularly the atypical connectivity involving the salience network and the temporoparietal junction, were consistently identified across two or more connectivity measures, reducing the likelihood that they represented false positives.

Consequently, the results were subject to certain constraints. To address these limitations, we plan to implement several targeted strategies in future research. First, we will expand recruitment efforts with a deliberate focus on enrolling more female participants to achieve a sex-balanced sample, enabling adequately powered sex-stratified analyses and sex-by-diagnosis interaction testing. Second, we intend to adopt a multisite collaborative recruitment approach, which may facilitate the inclusion of a more demographically diverse cohort, including a larger proportion of female individuals with ASD. Third, beyond simply increasing female enrollment, we will incorporate systematic assessments of sex-specific clinical phenotypes, including sex-normed measures of empathy and social cognition, to better characterize potential sex differences in the behavioral and neural manifestations of ASD. Finally, we intend to integrate advanced model-driven and data-driven techniques to enhance our understanding of the neural mechanisms of autism, identify more robust brain imaging biomarkers that account for sex as a biological variable, and refine tools for emotion recognition in children with and without ASD in the future.

## Conclusion

Our results suggested that DLSC could extract effective and robust brain ROI atlases. Functional connectomes with high differentiation power were mainly distributed in the brain networks of the SN, TPJ, and SBN. Individuals with ASD had lower empathy capability than control subjects, which may have been associated with dysfunctions of the salience and social brain networks.

## Data Availability

To protect study participant privacy, we don’t share research data publicly directly online. But Readers may contact the corresponding author for the original data after the paper is published. Requests to access these datasets should be directed to liyunb1989@163.com.
