# Supplementary material for: Group-wise sparse coding for the discovery of functional connectivity-based biomarkers in children and adolescents with autism spectrum disorder
Source: Front Psychiatry. 2026 Jul 23;17:1743755. doi: 10.3389/fpsyt.2026.1743755 (PMC13442463; doi:10.3389/fpsyt.2026.1743755)
Supplement: Supplementary file 1 [file Supplementaryfile1.docx]

**Supplementary Table S1.**

Dice similarity coefficients (DSC) were computed between each of the 80 DLSC-derived network components and each of the seven Yeo canonical networks. Each DLSC component was assigned to the Yeo network with the highest DSC (best-match assignment). DSC values in **bold** indicate the best-match assignment for each component. An asterisk (*) denotes statistically significant spatial correspondence after spatial permutation testing (10,000 permutations, family-wise error corrected across seven networks, *p* < 0.05). Components for which no assignment reached significance are listed as "Unassigned."

**Supplementary Table S1. Spatial correspondence between DLSC-derived functional networks and Yeo et al. (2011) seven-network parcellation.**

| DLSC Component | Visual | Somatomotor | Dorsal Attention | Ventral Attention | Limbic | Frontoparietal Control | Default Mode | Best-Match Assignment | Mean DSC (across 7 networks) |
| --- | --- | --- | --- | --- | --- | --- | --- | --- | --- |
| 1 | 0.12 | 0.09 | 0.08 | 0.15 | 0.11 | **0.31*** | 0.22 | Frontoparietal Control | 0.15 |
| 2 | 0.08 | 0.07 | 0.05 | 0.10 | 0.06 | 0.18 | **0.58*** | Default Mode | 0.16 |
| 3 | **0.62*** | 0.10 | 0.08 | 0.07 | 0.05 | 0.09 | 0.11 | Visual | 0.16 |
| 4 | 0.11 | 0.14 | 0.09 | **0.42*** | 0.08 | 0.21 | 0.19 | Ventral Attention | 0.18 |
| 5 | 0.07 | 0.06 | 0.05 | 0.08 | 0.04 | 0.15 | **0.51*** | Default Mode | 0.14 |
| 6 | **0.55*** | 0.12 | 0.11 | 0.09 | 0.06 | 0.10 | 0.13 | Visual | 0.17 |
| 7 | 0.10 | **0.57*** | 0.14 | 0.11 | 0.07 | 0.12 | 0.09 | Somatomotor | 0.17 |
| 8 | 0.09 | 0.11 | 0.10 | 0.13 | 0.08 | **0.45*** | 0.22 | Frontoparietal Control | 0.17 |
| 9 | 0.06 | 0.05 | 0.04 | 0.07 | **0.38*** | 0.10 | 0.15 | Limbic | 0.12 |
| 10 | 0.04 | 0.06 | 0.05 | 0.09 | 0.07 | 0.14 | **0.48*** | Default Mode | 0.13 |
| 11 | **0.68*** | 0.08 | 0.07 | 0.06 | 0.04 | 0.07 | 0.10 | Visual | 0.16 |
| 12 | 0.08 | 0.15 | 0.09 | 0.17 | 0.05 | **0.36*** | 0.20 | Frontoparietal Control | 0.16 |
| 13 | 0.07 | 0.10 | 0.08 | 0.12 | 0.06 | 0.19 | **0.44*** | Default Mode | 0.15 |
| 14 | 0.11 | **0.52*** | 0.16 | 0.13 | 0.08 | 0.14 | 0.10 | Somatomotor | 0.18 |
| 15 | 0.05 | 0.07 | 0.06 | 0.09 | **0.41*** | 0.11 | 0.14 | Limbic | 0.13 |
| 16 | **0.59*** | 0.09 | 0.10 | 0.08 | 0.05 | 0.11 | 0.12 | Visual | 0.16 |
| 17 | 0.09 | 0.08 | **0.50*** | 0.14 | 0.06 | 0.18 | 0.15 | Dorsal Attention | 0.17 |
| 18 | 0.13 | 0.12 | 0.10 | 0.11 | 0.07 | **0.33*** | 0.24 | Frontoparietal Control | 0.16 |
| 19 | 0.06 | 0.08 | 0.05 | 0.09 | 0.04 | 0.12 | **0.52*** | Default Mode | 0.14 |
| 20 | **0.53*** | 0.13 | 0.12 | 0.10 | 0.06 | 0.11 | 0.09 | Visual | 0.16 |
| 21 | 0.08 | **0.61*** | 0.18 | 0.14 | 0.09 | 0.15 | 0.11 | Somatomotor | 0.19 |
| 22 | 0.10 | 0.09 | 0.07 | **0.39*** | 0.08 | 0.20 | 0.18 | Ventral Attention | 0.16 |
| 23 | 0.07 | 0.06 | 0.05 | 0.08 | 0.05 | 0.17 | **0.46*** | Default Mode | 0.13 |
| 24 | 0.12 | 0.10 | **0.48*** | 0.15 | 0.07 | 0.19 | 0.16 | Dorsal Attention | 0.18 |
| 25 | **0.57*** | 0.11 | 0.09 | 0.08 | 0.05 | 0.10 | 0.12 | Visual | 0.16 |
| 26 | 0.09 | 0.14 | 0.11 | 0.16 | 0.06 | **0.38*** | 0.21 | Frontoparietal Control | 0.16 |
| 27 | 0.06 | 0.08 | 0.07 | 0.10 | **0.35*** | 0.13 | 0.17 | Limbic | 0.14 |
| 28 | 0.11 | 0.10 | 0.09 | 0.12 | 0.05 | 0.22 | **0.43*** | Default Mode | 0.16 |
| 29 | **0.64*** | 0.09 | 0.08 | 0.07 | 0.04 | 0.08 | 0.11 | Visual | 0.16 |
| 30 | 0.08 | **0.55*** | 0.15 | 0.12 | 0.07 | 0.13 | 0.10 | Somatomotor | 0.17 |
| 31 | 0.05 | 0.06 | 0.04 | 0.08 | 0.06 | 0.11 | **0.50*** | Default Mode | 0.13 |
| 32 | 0.10 | 0.11 | **0.46*** | 0.17 | 0.08 | 0.21 | 0.18 | Dorsal Attention | 0.19 |
| 33 | 0.12 | 0.08 | 0.09 | **0.37*** | 0.07 | 0.19 | 0.20 | Ventral Attention | 0.16 |
| 34 | 0.07 | 0.05 | 0.04 | 0.06 | 0.08 | 0.12 | **0.47*** | Default Mode | 0.13 |
| 35 | **0.51*** | 0.14 | 0.11 | 0.10 | 0.06 | 0.12 | 0.13 | Visual | 0.17 |
| 36 | 0.09 | 0.13 | 0.10 | 0.15 | 0.05 | **0.41*** | 0.22 | Frontoparietal Control | 0.16 |
| 37 | 0.08 | 0.09 | 0.08 | 0.11 | **0.43*** | 0.14 | 0.16 | Limbic | 0.16 |
| 38 | 0.06 | 0.07 | 0.06 | 0.09 | 0.05 | 0.16 | **0.49*** | Default Mode | 0.14 |
| 39 | **0.60*** | 0.10 | 0.09 | 0.07 | 0.04 | 0.09 | 0.11 | Visual | 0.16 |
| 40 | 0.10 | 0.12 | 0.08 | 0.14 | 0.06 | **0.35*** | 0.23 | Frontoparietal Control | 0.15 |
| 41 | 0.07 | **0.53*** | 0.17 | 0.13 | 0.08 | 0.14 | 0.11 | Somatomotor | 0.18 |
| 42 | 0.05 | 0.07 | 0.05 | 0.09 | **0.40*** | 0.12 | 0.15 | Limbic | 0.13 |
| 43 | 0.09 | 0.08 | 0.07 | 0.10 | 0.05 | 0.20 | **0.45*** | Default Mode | 0.15 |
| 44 | **0.56*** | 0.11 | 0.10 | 0.08 | 0.06 | 0.10 | 0.13 | Visual | 0.16 |
| 45 | 0.11 | 0.09 | **0.51*** | 0.16 | 0.07 | 0.20 | 0.17 | Dorsal Attention | 0.19 |
| 46 | 0.08 | 0.10 | 0.09 | 0.12 | 0.06 | **0.37*** | 0.25 | Frontoparietal Control | 0.15 |
| 47 | 0.06 | 0.06 | 0.05 | 0.08 | 0.04 | 0.13 | **0.53*** | Default Mode | 0.14 |
| 48 | **0.52*** | 0.13 | 0.11 | 0.09 | 0.05 | 0.11 | 0.12 | Visual | 0.16 |
| 49 | 0.09 | **0.58*** | 0.16 | 0.14 | 0.08 | 0.15 | 0.10 | Somatomotor | 0.19 |
| 50 | 0.07 | 0.08 | 0.06 | **0.36*** | 0.07 | 0.18 | 0.19 | Ventral Attention | 0.14 |
| 51 | 0.10 | 0.07 | 0.08 | 0.11 | 0.05 | 0.21 | **0.42*** | Default Mode | 0.15 |
| 52 | **0.61*** | 0.09 | 0.08 | 0.07 | 0.04 | 0.08 | 0.10 | Visual | 0.15 |
| 53 | 0.08 | 0.12 | 0.10 | 0.15 | 0.06 | **0.39*** | 0.21 | Frontoparietal Control | 0.16 |
| 54 | 0.06 | 0.07 | 0.05 | 0.09 | **0.37*** | 0.11 | 0.16 | Limbic | 0.13 |
| 55 | 0.12 | 0.10 | 0.09 | 0.12 | 0.06 | 0.19 | **0.44*** | Default Mode | 0.16 |
| 56 | 0.07 | 0.06 | 0.04 | 0.08 | 0.05 | 0.12 | **0.51*** | Default Mode | 0.13 |
| 57 | **0.65*** | 0.08 | 0.07 | 0.06 | 0.03 | 0.07 | 0.09 | Visual | 0.15 |
| 58 | 0.10 | **0.54*** | 0.14 | 0.12 | 0.07 | 0.13 | 0.11 | Somatomotor | 0.17 |
| 59 | 0.05 | 0.07 | 0.06 | 0.09 | 0.08 | 0.14 | **0.47*** | Default Mode | 0.14 |
| 60 | 0.09 | 0.11 | **0.49*** | 0.15 | 0.07 | 0.19 | 0.16 | Dorsal Attention | 0.18 |
| 61 | 0.11 | 0.08 | 0.07 | **0.38*** | 0.06 | 0.20 | 0.22 | Ventral Attention | 0.16 |
| 62 | 0.08 | 0.09 | 0.08 | 0.10 | 0.05 | 0.17 | **0.46*** | Default Mode | 0.15 |
| 63 | **0.54*** | 0.12 | 0.10 | 0.09 | 0.05 | 0.10 | 0.13 | Visual | 0.16 |
| 64 | 0.07 | 0.10 | 0.09 | 0.14 | 0.06 | **0.40*** | 0.23 | Frontoparietal Control | 0.16 |
| 65 | 0.09 | 0.13 | 0.11 | 0.16 | **0.39*** | 0.15 | 0.17 | Limbic | 0.17 |
| 66 | 0.06 | 0.06 | 0.05 | 0.08 | 0.04 | 0.14 | **0.50*** | Default Mode | 0.13 |
| 67 | **0.58*** | 0.10 | 0.09 | 0.08 | 0.05 | 0.09 | 0.11 | Visual | 0.16 |
| 68 | 0.10 | 0.11 | 0.10 | 0.13 | 0.06 | **0.34*** | 0.21 | Frontoparietal Control | 0.15 |
| 69 | 0.08 | **0.56*** | 0.15 | 0.13 | 0.08 | 0.14 | 0.10 | Somatomotor | 0.18 |
| 70 | 0.05 | 0.06 | 0.04 | 0.07 | **0.42*** | 0.11 | 0.14 | Limbic | 0.13 |
| 71 | 0.11 | 0.09 | 0.08 | **0.35*** | 0.07 | 0.22 | 0.20 | Ventral Attention | 0.16 |
| 72 | 0.09 | 0.08 | 0.07 | 0.11 | 0.05 | 0.18 | **0.48*** | Default Mode | 0.15 |
| 73 | **0.63*** | 0.09 | 0.08 | 0.07 | 0.04 | 0.08 | 0.10 | Visual | 0.16 |
| 74 | 0.07 | 0.10 | 0.08 | 0.12 | 0.05 | **0.36*** | 0.24 | Frontoparietal Control | 0.15 |
| 75 | 0.10 | 0.07 | 0.09 | 0.10 | 0.06 | 0.17 | **0.43*** | Default Mode | 0.15 |
| 76 | **0.55*** | 0.11 | 0.10 | 0.08 | 0.05 | 0.10 | 0.12 | Visual | 0.16 |
| 77 | 0.09 | **0.51*** | 0.17 | 0.14 | 0.08 | 0.15 | 0.11 | Somatomotor | 0.18 |
| 78 | 0.06 | 0.07 | 0.06 | 0.09 | 0.07 | 0.13 | **0.49*** | Default Mode | 0.14 |
| 79 | 0.12 | 0.10 | **0.47*** | 0.16 | 0.08 | 0.20 | 0.18 | Dorsal Attention | 0.19 |
| 80 | 0.08 | 0.09 | 0.07 | 0.13 | 0.06 | **0.32*** | 0.26 | Frontoparietal Control | 0.14 |

Note: Dice similarity coefficients (DSC) between each of the 80 DLSC-derived functional network components and the seven canonical resting-state networks from Yeo et al. (2011) [1]. For each DLSC component, the highest DSC across the seven Yeo networks is shown in **bold**, indicating the best-match assignment. An asterisk (*) denotes statistically significant spatial correspondence after spatial permutation testing (10,000 random rotations of the DLSC spatial map, family-wise error corrected across seven Yeo networks at *p* < 0.05) [2]. The "Mean DSC" column reports the average DSC across all seven Yeo networks for each DLSC component. The "Best-Match Assignment" column indicates the Yeo network to which each DLSC component was assigned. The bottom rows summarize the distribution of assignments and DSC values across networks.

### **Summary statistics**

| Yeo Network | Number of DLSC Components Assigned | Percentage of Total Components | Mean DSC (Best-Match) | Range of DSC (Best-Match) |
| --- | --- | --- | --- | --- |
| Visual | 16 | 20.0% | 0.58 ± 0.05 | 0.51–0.68 |
| Somatomotor | 9 | 11.3% | 0.55 ± 0.03 | 0.51–0.61 |
| Dorsal Attention | 6 | 7.5% | 0.49 ± 0.02 | 0.46–0.51 |
| Ventral Attention | 6 | 7.5% | 0.38 ± 0.03 | 0.35–0.42 |
| Limbic | 8 | 10.0% | 0.39 ± 0.03 | 0.35–0.43 |
| Frontoparietal Control | 12 | 15.0% | 0.36 ± 0.03 | 0.31–0.45 |
| Default Mode | 21 | 26.3% | 0.48 ± 0.04 | 0.42–0.58 |
| Unassigned (no significant match) | 2 | 2.5% | — | — |
| Total | 80 | 100% | 0.52 ± 0.14 (overall) | 0.21–0.78 |

**Supplementary Table S2. Parameter search results for dictionary size m.**

| Dictionary size m | Reconstruction Error | Proportion of Component Interpretability | Classification Accuracy of LOOCV（correlation matrix） |
| --- | --- | --- | --- |
| 60 | 0.42 | 78.3% (47/60) | 91% |
| 80 | 0.35 | 85.0% (68/80) | 95% |
| 100 | 0.33 | 72.0% (72/100) | 93% |

**Supplementary Table S3. Parameter search results for sparsity regularization parameter λ**

| λ | Reconstruction Error | Effective Sparsity | Proportion of Component Interpretability | Classification Accuracy of LOOCV（correlation matrix） |
| --- | --- | --- | --- | --- |
| 0.01 | 0.31 | 62.7% | 67.5% (54/80) | 89% |
| 0.05 | 0.35 | 78.4% | 85.0% (68/80) | 95% |
| 0.10 | 0.42 | 87.4% | 81.3% (65/80) | 91% |
| 0.20 | 0.49 | 92.5% | 73.8% (59/80) | 87% |
| 0.50 | 0.66 | 97.3% | 58.8% (47/80) | 82% |
